# Association between Stock Market Gains and Losses and Google Searches

**DOI:** 10.1371/journal.pone.0141354

**Published:** 2015-10-29

**Authors:** Eli Arditi, Eldad Yechiam, Gal Zahavi

**Affiliations:** Faculty of Industrial Engineering and Management, Technion–Israel Institute of Technology, Haifa, 3200001, Israel; Uppsala University, SWEDEN

## Abstract

Experimental studies in the area of Psychology and Behavioral Economics have suggested that people change their search pattern in response to positive and negative events. Using Internet search data provided by Google, we investigated the relationship between stock-specific events and related Google searches. We studied daily data from 13 stocks from the Dow-Jones and NASDAQ100 indices, over a period of 4 trading years. Focusing on periods in which stocks were extensively searched (Intensive Search Periods), we found a correlation between the magnitude of stock returns at the beginning of the period and the volume, peak, and duration of search generated during the period. This relation between magnitudes of stock returns and subsequent searches was considerably magnified in periods following negative stock returns. Yet, we did not find that intensive search periods following losses were associated with more Google searches than periods following gains. Thus, rather than increasing search, losses improved the fit between people’s search behavior and the extent of real-world events triggering the search. The findings demonstrate the robustness of the attentional effect of losses.

## Introduction

Internet search engines have become an important tool for information gathering. Consequentially, analyzing data collected by major search engines such as Google may shed light on relations between real-world events and people’s information search behavior. For instance, previous studies have shown that search-term volumes were correlated with events such as surges of unemployment [1] and reports of flu infections [2]. Likewise, query volumes of company names were found to be correlated with their stock-market trading volumes [3,4] and volatility [5]. In addition, some studies have suggested that specific search queries may even anticipate subsequent stock market price changes [6,7,8]. For instance, a relative increase in search of the word ‘debt’ tended to precede stock market decline [6]. In the current study, rather than testing the effect of search on stock-price dynamics, we investigated how search for stock-related information is affected by market events, as reflected by stock price changes [9]. Focusing on time periods when specific stocks were extensively searched (Intensive Search Periods; or ISPs), we evaluated the relation between stock price changes at the beginning of the period and search for information concerning the stock during the period. In particular, we examined whether the relation between the extent of stock price changes and subsequent volumes of searches is stronger for negative price movements than for positive ones, reflecting greater sensitivity of searches to negative events.

Studies in Psychology and Behavioral Economics have suggested that people respond asymmetrically to positive and negative events [10,11,12]. Specifically, under the idea of loss aversion a person’s losses are over-weighted compared to equivalent gains [10]. Most recently, the generality of loss aversion in empirical studies has been questioned [13]. However, even in the absence of loss aversion as classically defined, losses were found to increase cognitive effort, deliberation time, and performance compared to respective gains [13,14,15], and this has been explained as due to greater task attention (i.e., enhanced vigilance) with losses [13]. In other studies, losses were also found to increase search among relevant choice alternatives [16,17,18]. For example, Huber et al. (2014) [18] showed that the number of questions asked by participants concerning a risky situation they were about to deal with, was substantially higher when the situation was framed negatively than when it was framed positively. These findings have led us to predict that the extent of searches for stock-specific information would be more sensitive to the amount of losses sustained by a given stock than to the amount of gains; as would be evident by a higher correlation between the size of a loss (compared to a gain) and the subsequent volume of searches.

Note that some scholars have suggested that psychological biases, and particularly the endowment effect which is often attributed to loss aversion, do not affect behavior in experiential market settings [19. 20]. However, we suggest that vis-à-vis the robustness of stock market pricing behavior to certain biases involving losses (cf. [21]), individuals who are responding to market-related information exhibit different information search patterns in response to gains and losses. These disparate information search patterns do not influence the mean stock prices since prices often converge to an efficient equilibrium [9, 22], but they nevertheless constitute an important outcome variable that can be predicted (and potentially exploited).

In order to evaluate this prediction, we retrieved daily data about U.S. Google users searching for U.S.-traded stocks. We downloaded from *Google Trends* the daily U.S. Search Volume Index (SVI) for 127 ticker symbols of the stocks included in the Dow Jones index and NASDAQ100 index, for the period of Jan 2009 to Nov 2013. After filtering out stocks for incomplete data provided by Google, and search terms that are completely disassociated with stock-related searches (e.g., due to a ticker symbol being identical to another commonly searched word; see Method section for details), we remained with 13 reliable daily search time-series. Therefore, our analysis included 13 stocks (AAPL, ADP, BIDU, CSCO, GOOG, INTC, JNJ, JPM, MSFT, PFE, WMT, XOM, and YHOO) and 1237 trading days (a total of 16,081 observations of daily SVI and stocks prices). In order to study bursts of searches for a given stock (which are not necessarily everyday events), our study focused on Intensive Search Periods (ISPs). ISPs were defined according to an abnormally high search volume. We investigated the relationship between the magnitude and valence (i.e., positivity vs. negativity) of the daily stock return at the beginning of the ISP and properties of search during this time period, i.e., its volume, peak level, and duration.

## Method

### Data collection

Choosing which search terms to download is a crucial ingredient of every study utilizing the Google Trends tool. In identifying a search for a stock, using a company name may be problematic [7], since the user may google the company name for reasons unrelated to investing (e.g., “EBAY” for online shopping), or for reasons not related to the company at all (e.g., “Apple”). Furthermore, the same firm may be googled using different phrases (e.g., “American Airlines” or “AA” or “AMR Corp” or “AMR”). On the other hand, stock tickers are often uniquely assigned [4]. Therefore, we focused on searches for tickers symbols.

Since Google Trends provides SVI data for various regions of the world, one can also constrain the relevant region. Because of our focus on U.S. exchange traded stocks, we restricted the dataset for searches in the U.S. This choice is consistent with the results of Preis et al. (2013) [3] who tried to build profitable trading strategies for the U.S. stock-market based on Google Trends, and found that strategies based on search volume data for U.S. users were more successful than strategies based on global search data.

Downloading daily SVIs raises another challenge: Google Trends provides daily SVIs only for short time periods of 3 months (or less), and each resulting time series is scaled by the time series maximum SVI in that quarter. In order to create a long unified time series of a few years for each search-term, we employed a web crawling program that downloads, for each ticker, the SVI of overlapping two-month periods in ascending order (e.g., first 01/2009-02/2009, then 02/2009-03/2009, etc.). Although each period is normalized (the maximum value of each period is always 100), the common month of each two consequent periods enables calculating the multiplication factor needed for scaling and joining all of the short-period data into one long and consistent time series per search-term. To verify that this transformed data is consistent with the longer series data, we downloaded the weekly data for the ADP, GOOG, and AAPL tickers for Jan2009-Nov2013 and compared it to our daily data, by transforming the daily vector to a weekly vector (i.e., summing the 7 days in each week). The correlations between the 2 time series were 0.99, 0.99, and 0.96 respectively.

Using our crawling program, we tried to download daily SVI data for each of the stocks included in the Dow-Jones and NASDAQ100 indices, over the period of 01/2009–11/2013. Unfortunately, for search terms with low popularity, Google provides breakdown of its data only by week instead of by day. Out of the 30 stocks of the Dow Jones index, only 25 had daily data for the whole time period, and out of the 100 stocks of NASDAQ100, complete daily data was available for only 26 tickers. The total number tickers for which SVI data was collected was therefore 48 (because of overlaps between the indexes).

Examining the search data of the 48 tickers, we noticed that the SVI data may still not specifically capture searches related to the respective stocks. Some of the tickers had generic meaning (e.g., “CAT”, “FAST”, or “VOD”) while others were identical to the company name (e.g., “IBM” or “EBAY”). Some studies manually went through their sample stock tickers and flagged supposedly noisy tickers [4,7]. We developed an objective filtering approach using the stock’s trading volume. It seems reasonable that there will be a positive association between SVI and trading-volume of the stock, since at times when new information about a company is revealed, both trading and search-interest will increase. Indeed, previous studies have found that SVI for stock tickers is correlated with the trading volume of these stocks, both at a weekly [6] and daily [4] resolution. In order to filter out tickers with noisy SVI, we calculated the correlation between trading-volume changes and SVI changes for each ticker, on weekly data (See Table A in S1 Text). Tickers with positive relationship and r^2^ > 0.1 (our chosen cutoff) were considered as less noisy. Out of our 48 stock tickers, only 13 search-terms were categorized in this group. We find that this subgroup of tickers excludes those with generic meanings and tickers identical to the company name, which supports the validity of our filtering approach. Our analysis therefore focuses on these 13 tickers. For the remaining tickers, the r^2^ of the relation between SVI and volume dropped quite rapidly to near zero (see Table A in S1 Text), suggesting that search for these terms was independent of events relating to the relevant stock.

### Data processing

In order to identify ISPs in each search day *t*, we needed to calculate when the SVI is abnormally high. Following Da et al (2011) [7], we define our key variable of interest, abnormal SVI (ASVI), as:

$$ASVI_{t}=\ln(SVI_{t})-\text{median}\left[\ln(SVI_{t}),\ln(SVI_{t-1}),\ldots \ln(SVI_{t-59})\right],$$ (1)

The ASVI index approximates the relative daily SVI percent change, while neglecting outliers, namely extreme values and low-frequency seasonality. A large positive ASVI indicates increased search concerning the stock in a given day.

**Fig 1:**
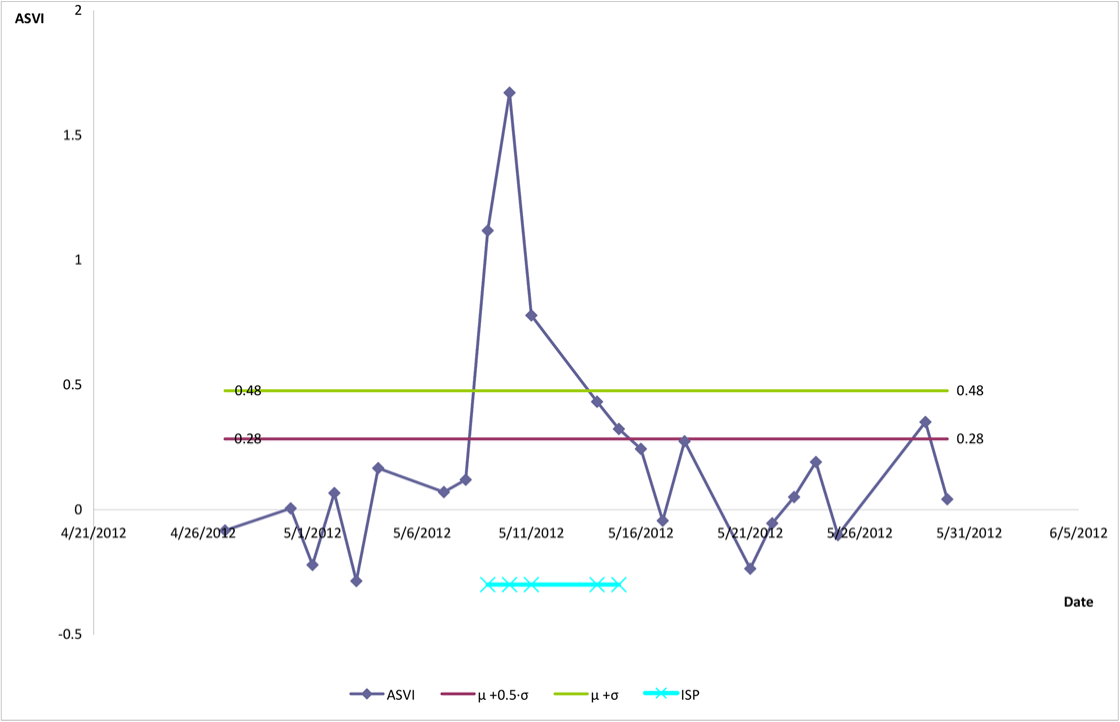
Relevant time series of Abnormal Search Volume Index (ASVI), for the Cisco stock symbol “CSCO”. The upper threshold (μ+σ) and the lower threshold (μ+0.5·σ) are noted. An Intensive Search Period (ISP) is specified from where the ASVI crosses and stays above the upper threshold until it crosses the lower threshold.

For each stock *i*, we calculated the ASVI mean (μ_*i*_) and standard-deviation (σ_*i*_) for the whole period. An ISP was defined as the time period when the ASVI in a given stock crosses and stays above an upper threshold (*t* = 0) until it crosses a lower threshold. We defined the upper threshold as μ_*i*_+σ_*i*_ and the lower threshold as μ_*i*_+0.5*σ_*i*_. The upper threshold is naturally higher because it captures the emergence of an extreme search event, whereas the lower threshold is lower because it marks the end of this event (we also evaluated other thresholds, see S2 Text). This set of consecutive days represents a steep surge in investors’ search behavior, followed by a consistent decay until the normal ASVI level is reached. Fig 1 presents an example of a single ISP.

We identified all ISPs from the examined stocks, and tagged each ISP as “positive” or “negative”, according to the sign of the daily stock-return at the beginning of the period, i.e., the stock price change compared to the previous day. Overall, 946 ISPs were observed, about 44% of which were negative.

For each ISP, we specified the following properties:

1. *ISP Peak*: The peak level of search reached at the beginning of the period. Interestingly, in many cases the peak ASVI day does not occur at *t* = 0 of the event (the first day of the ISP) but at *t* = 1. The main reason for that is the timing of important announcements: in many cases, a company announces important updates after the stock market is closed. The evening’s searches are counted at *t* = 0 of the event, but the ASVI peak takes place only at *t* = 1, when announcement-contingent trading takes place. Consequentially, we defined the ASVI peak as the maximum of the first 2 days of the period.
2. *ISP Sum*: The total amount of search generated for the period (summed across all period days).
3. *ISP Duration*: The length (in days) of the period.
4. *Absolute Stock Return*: The absolute log difference between the stock price on the peak-ASVI day and the previous day. We calculated the stock log-return as log(*p*_*t*_) − log(*p*_t-1_), where *p*_*t*_ is the stock closing price.
  We examined the relation between the absolute stock return at the peak-ASVI day and its Google search properties. We further checked whether the stock return movements can also *predict* ASVI on the days following the ASVI peak. To this end, we defined the following additional properties:
5. *ISP Post-Peak Sum*: The total amount of search during an ISP, starting from the day after the peak-ASVI day.
6. *ISP Post-Peak Duration*: The length (in days) of the ISP, counting from the day after the peak-ASVI day.

The data from various stocks and various ISPs was submitted to regression analyses, with each stock contributing a set of ISPs. Each search property was regressed on *Absolute Stock Return*. Additionally, in order to compare positive and negative ISPs, we defined a dummy variable IsNeg which equals 1 for negative and 0 for positive periods (i.e., *p*_*t*_ < *p*_t-1_ or *p*_*t*_ > *p*_t-1_, respectively). Three sets of regressions were run: 1) The main tests, which used *Absolute Stock Return* and IsNeg as predictors of the three search indices. 2) Planned contrast tests in which positive and negative events were separately evaluated. In each of these tests, *Absolute Stock Return* served as predictor. 3) Tests examining only the effect of valence in which IsNeg was the only predictor (these were supplemented by simple t-tests across all ISPs as a robustness check). In order to take into consideration the different stocks, we used cluster sample regression, which corrects the standard errors for correlations within tickers, and therefore provides accurate estimates of standard error while not reducing statistical power.

## Results

**Fig 2:**
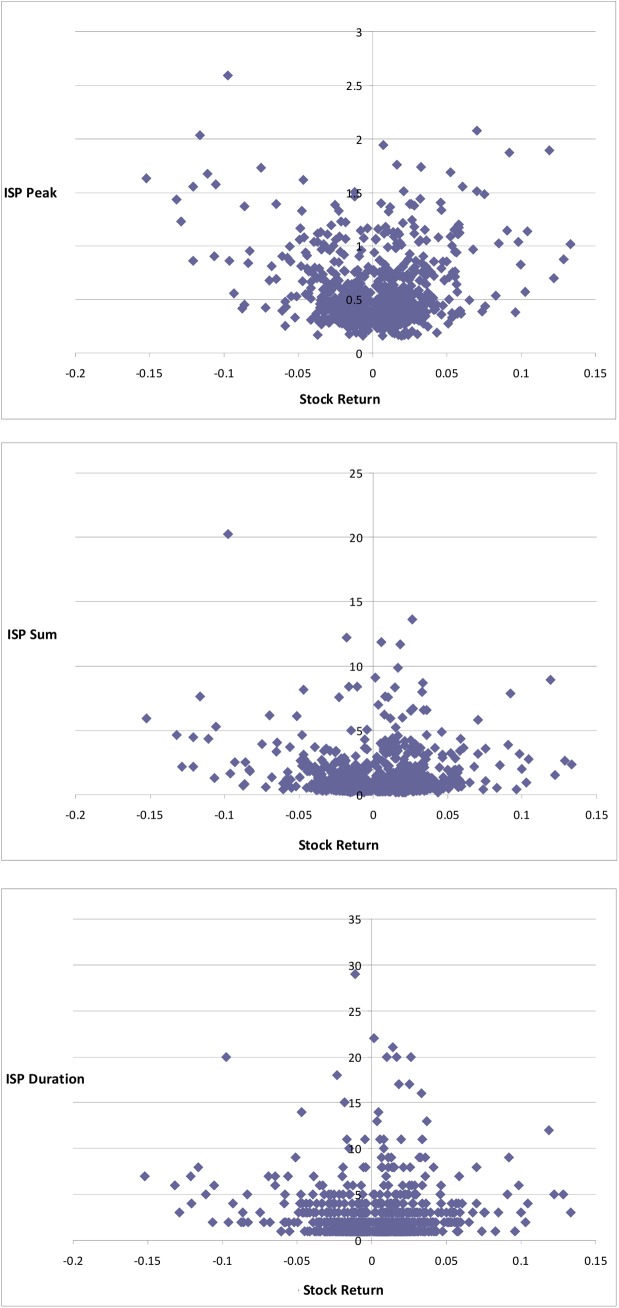
Scatter plots of the stock return at the peak ASVI and the three search indices.

Fig 2 shows the individual scatter plots of the relations between stock returns at the peak ASVI and the three search variables. The figure hints that these relations were somewhat different for positive and negative ISPs. Table 1 shows the regression results for the three dependent variables. As indicated in the table, there was a significant correlation between the magnitude of stock returns at the beginning of the period and the search volume, which was particularly evident for *ISP Peak* and *ISP Sum*.

**Table 1:** Regressions of *ISP Peak*, *ISP Sum*, and *ISP Duration* with *Absolute Stock Return* as predictor.

* *	*ISP Peak*	*ISP Sum*	*ISP Duration*
Intercept	0.41	0.96	2.32
IsNeg	-0.06 (0.005)	-0.49 (0.014)	-0.68 (0.021)
Absolute Stock Return	6.18 (0.001)	18.56 (0.001)	18.59 (0.022)
Absolute Stock Return × IsNeg	1.86 (0.041)	13.71 (0.023)	14.71 (0.029)
F (Model)	13.81 (< .001)	6.28 (0.008)	7.83 (0.004)
r^2^	0.25	0.11	0.05

Note: Estimated regression coefficients and p-values in parentheses, followed by the results of the model’s F test and explained variance (r^2^). The sample size was 947 for all three variables. The dummy variable IsNeg equaled 1 if the daily stock return at the beginning of the period was negative and 0 if not.

However, there was also an interaction between *Absolute Stock Return* and IsNeg in all of the three search volume variables, marking a difference in those relations for positive and negative ISPs. To further examine the source of this difference, we conducted planned contrast analyses that separately evaluated positive and negative periods. Examination of the results, summarized in Table 2, revealed a (consistent) higher r^2^ in the regressions for negative periods. For example, for *ISP Peak*, r^2^ was 0.35 for negative ISPs versus 0.16 for positive periods; for *ISP Sum*, r^2^ was 0.19 for negative versus only 0.04 for positive periods; a smaller difference in the same direction emerged for *ISP Duration*. This implies that the association between stock return at the beginning of an ISP and search volume during the period was stronger following loss events, particularly for *ISP Peak* and *ISP Sum*. Another finding in Table 1 was a negative main effect of IsNeg, indicating that the intercept of the relation was higher for gains than for losses. We shall later revisit this negative effect. Importantly, it does not necessarily imply that more searches were made after gains than after losses.

**Table 2:** Regressions of *ISP Peak*, *ISP Sum*, and *ISP Duration*, separately for positive and negative Intensive Search Periods.

	*ISP Peak*		*ISP Sum*		*ISP Duration*	
	Positive ISP	Negative ISP	Positive ISP	Negative ISP	Positive ISP	Negative ISP
Intercept	0.41	0.36	0.96	0.47	2.32	1.64
Absolute Stock Return	6.18 (0.001)	8.04 (< .001)	18.56 (0.001)	32.27 (0.001)	18.59 (0.022)	33.31 (0.004)
r^2^	0.16	0.35	0.04	0.19	0.02	0.09

Note: Estimated regression coefficients and p-values in parentheses, followed by the explained variance (r^2^). The sample size was 534 for the positive ISP regressions and 413 for the negative ISP regressions.

Additionally, we examined whether *Absolute Stock Return* can also predict ASVI on the days following the peak of the search period. The findings are presented in Table 3. The results indicated that stock return at the beginning of the period did not significantly predict the subsequent cumulative ASVI and the duration of the period. Also, as previously there was a significant interaction between the effect of stock returns and the dummy variable IsNeg, marking a difference between the predictive values of negative versus positive events. Planned contrast analyses were conducted to separately examine positive and negative ISPs (see Table 4). The results showed that predictive power was again considerably stronger for negative than for positive periods (e.g., for *ISP Post-Peak Sum*, r^2^ = 0.09 for negative compared to r^2^ = 0.01 for positive ISPs).

**Table 3:** Regressions of *ISP Post-Peak Sum* and *ISP Post-Peak Duration* with *Absolute Stock Return* as predictor.

* *	*ISP Post-Peak Sum*	*ISP Post-Peak Duration*
Intercept	0.52	1.20
IsNeg	-0.39 (0.027)	-0.62 (0.024)
Absolute Stock Return	7.76 (0.062)	13.85 (0.051)
Absolute Stock Return × IsNeg	10.55 (0.040)	13.76 (0.031)
F (Model)	4.07 (0.032)	6.23 (0.008)
r^2^	0.05	0.03

Note: Estimated regression coefficients and p-values in parentheses, followed by the results of the model’s F test and explained variance (r^2^). The sample size was 947 for both variables. The dummy variable IsNeg equaled 1 if the daily stock return at the beginning of the period was negative and 0 if not.

**Table 4:** Regressions of *ISP Post-Peak Sum* and *ISP Post-Peak Duration*, separately for positive and negative Intensive Search Periods.

	*ISP Post-Peak Sum*		*ISP Post-Peak Duration*	
	Positive ISP	Negative ISP	Positive ISP	Negative ISP
Intercept	0.52	0.14	1.20	0.58
Absolute Stock Return	7.76 (0.062)	18.31 (0.006)	13.85 (0.05)	27.61 (0.001)
r^2^	0.01	0.09	0.01	0.06

Note: Estimated regression coefficients and p-values in parentheses. The sample size was 534 for the positive ISP regressions and 413 for the negative ISP regressions.

We also examined the relation between the daily stock return at the peak-ASVI point and subsequent stock price change throughout the ISP. This clarifies whether the studied time periods confirm to the efficient market hypothesis [9, 22], which asserts that in financial markets security prices reflect all the available information; and are thus not anomalous in this sense. The results showed no correlation between daily stock returns at the origin and subsequent stock price changes throughout the ISP (for negative ISPs: r^2^ = 0.009 for positive ones: r^2^ = 0.001).

To assess the robustness of our results we conducted regression analyses with different levels of upper thresholds (defining where an ISP begins) of μ_*i*_+0.5*σ_*i*_, μ_*i*_+2*σ_*i*_, and μ_*i*_+3*σ_*i*_ for each stock *i*. The findings are summarized in the supplementary information section (S2 Text). Effect sizes were smaller than in our main analysis (cf. Tables 1 and 2), due to the somewhat noisier data (for μ_*i*_+0.5) and smaller number of observations (for μ_*i*_+2*σ_*i*_ and above). However, the most consistent result of this analysis, for all of the different evaluated thresholds and search-related dependent variables, was a much higher r^2^ in the regressions for negative periods compared to positive periods. This stresses that in negative periods stock returns were more predictive of subsequent Internet search volumes. We also verified that the results are not due to the dynamics of but a few stocks: Higher r^2^ for negative periods was found in an investigation of the majority of the studies stocks (see Table C in S1 Text); the results were also not driven by the distribution of the data (see S3 Text).

Finally, we also evaluated whether more extensive Google searches take place in negative than in positive ISPs (namely, more searches follow a negative market event). For this purpose, we examined the effect of the dummy variable IsNeg on the three search indices. As shown in Table 5, cluster sample regressions showed no significant correlation between IsNeg and any of the search-related indices. This effect was replicated in t-tests conducted across the different tickers: there was no significant difference between positive and negative periods for *ISP Peak* (t(945) = 0.42, p = .67), *ISP Sum* (t(945) = 1.44, p = 0.15), and *ISP Duration* (t(945) = 1.82, p = .07) (there was also no difference in the standard deviation of the search indices for positive and negative ISPs: ISP Peak Pos: SD = 0.31, Neg: SD = 0.31; ISP Sum Pos: SD = 1.73, Neg: SD = 1.68; ISP Duration Pos: SD = 2.91, Neg: SD = 2.59). This was not due to a confound with the absolute return at the beginning of the ISP, since the mean absolute return at the origin was almost identical for positive and negative ISPs (M = 0.021, 0.022 respectively). Note also, that this finding is compatible with the negative effect of IsNeg in Table 1. As noted above, because the analysis presented in Table 1 controlled for the interaction between absolute stock returns and IsNeg, the observed main effect of IsNeg was not indicative of the total effect of this variable (but instead indicated the different intercepts for negative versus positive ISPs). The absence of a main effect of valence implies that our key findings above are not due to increased search following negative events, but instead reflect greater sensitivity to differences between negative events than between positive events.

**Table 5:** Regressions of *ISP Peak*, *ISP Sum*, and *ISP Duration* with IsNeg as predictor.

* *	*ISP Peak*	*ISP Sum*	*ISP Duration*
Intercept	0.54	1.35	2.71
IsNeg	-0.01 (0.636)	-0.16 (0.279)	-0.33 (0.130)
Observations	947	947	947
r^2^	0.0002	0.002	0.003

Note: Estimated regression coefficients and p-values in parentheses. The dummy variable IsNeg equaled 1 if the daily stock return at the beginning of the period was negative and 0 if not.

## Discussion

Focusing on periods where there was an atypical increase of search in Google (ISPs), we found an effect of the absolute size of the daily stock return at the beginning of the period on peak, volume, and duration of intensive search periods. In addition, as predicted, for negative daily returns the correlations between returns and the search indices were considerably higher. However, we did not find that Internet searches tended to increase (or decrease) following negative daily returns. Thus, rather than reflecting increased search behavior following losses [16,17,18], the current findings demonstrate better fit between the extremity of the market event and the resultant behavioral search for loss events. The findings are thus consistent with the idea that losses increase attention [13], and specifically with the notion that people are more sensitive to differences between losses than to differences between respective gains [23,24,25].

One might argue that negative changes in stock prices are perceived as opportunities rather than mishaps. However, this seems inconsistent with studies showing that despite the null autocorrelations commonly observed between returns in subsequent days [9, 22], investors typically err in the direction of assuming positive autocorrelations [26, 27], errors which are then often exploited by short sellers [28]. Hence, for many investors a loss is perceived as predictive of future losses, rather than opportunities. Nevertheless, it is left for future studies to examine the nature of the exact psychological mechanisms leading investors to be more sensitive to negative stock returns in subsequent search efforts.

Another interesting direction is to examine whether the disparate effect of gains and losses on information search influences stock-market price dynamics. In this respect, while individuals’ search strategies might not affect the mean stock returns, they may affect the volatility (i.e., variance) of the returns. In fact, it is well known that bursts of volatility are more sensitive to the size of losses than to the size of respective gains, a phenomenon known as the “leverage effect” [29]. The economic reasons for this phenomenon remain in dispute. The common explanation attributes the phenomenon to changes in financial leverage of the firm, or debt-to-equity ratios [30,31]. However, recent studies question this explanation [32,33,34,35]. An alternative proposal is that the leverage effect is due to an asymmetric correlation between returns and subsequent trading volumes due to the varying psychological reaction of investors to gains and losses [36,37]. Consistent with this notion, recent studies that evaluated the leverage effect in various countries, found that the effect is most pronounced in markets with high participation of private investors [38,39]. The current findings are consistent with the suggestion that investors exhibit disparate sensitivity to positive and negative returns. Additionally, there might also be a causal path between search for information (on the web and in other places) and volume changes. Internet search of stock-related information on Google was found to be positively associated with trading volume and volatility of stock returns [3,4,5]. Thus, the current findings provide complementary evidence suggesting that the source of the leverage effect lies in the distinct psychological sensitivity of individual investors to losses and gains.

In conclusion, our findings indicate that in order to predict the extent of periods of intensive search in Google one cannot rely on absolute value of stock returns at the beginning of the period alone. Instead, the valence of the returns plays an important part in this relation. The size of positive returns provides rather poor predictive power for subsequent searches; while the size of negative returns more strongly predicts the extent of subsequent search. This emerges even though search volume is similar following positive and negative returns. These findings join a growing corpus of studies showing that sensitivity of human behavior to differences between external events increases when the task they are facing involves losses [23,24,25]. Here we highlight the phenomenon that even in an efficient market setting the sensitivity of information search to the size of relevant events appears to be higher following losses than gains.

## Supporting Information

S1 Text Additional information on the stock tickers. (DOC)

S2 Text Examination of different criteria for Intensive Search Periods. (DOC)

S3 Text Examination of differences between correlations in randomly permuted conditions. (DOC)
