# Supplementary material for: Association between Stock Market Gains and Losses and Google Searches
Source: PLoS One. 2015 Oct 29;10(10):e0141354. doi: 10.1371/journal.pone.0141354 (PMC4626086; doi:10.1371/journal.pone.0141354)
Supplement: S1 Text — (DOC) [file pone.0141354.s001.doc]

Association between Stock Market Gains and Losses and Google Searches

S1. Additional information on the stock tickers

Table A. Correlations of trading-volume changes and SVI changes on weekly data, for 48 stock tickers. Tickers with a positive relation (r > 0) and r^2^ > 0.1 were included in the analysis.

| *Ticker* | *r* | *r^2^* |
| --- | --- | --- |
| CSCO | 0.769 | 0.592 |
| AAPL | 0.761 | 0.579 |
| MSFT | 0.647 | 0.419 |
| INTC | 0.619 | 0.383 |
| BIDU | 0.617 | 0.381 |
| JPM | 0.612 | 0.375 |
| GOOG | 0.586 | 0.343 |
| WMT | 0.497 | 0.247 |
| PFE | 0.426 | 0.181 |
| YHOO | 0.425 | 0.181 |
| JNJ | 0.379 | 0.143 |
| XOM | 0.364 | 0.132 |
| ADP | 0.340 | 0.116 |
| HD | -0.327 | 0.107 |
| IBM | 0.290 | 0.084 |
| GS | 0.276 | 0.076 |
| GE | 0.263 | 0.069 |
| COST | 0.257 | 0.066 |
| SIRI | 0.225 | 0.051 |
| DD | 0.223 | 0.050 |
| DIS | 0.208 | 0.043 |
| MAR | -0.199 | 0.040 |
| MCD | 0.194 | 0.038 |
| CVX | 0.190 | 0.036 |
| WYNN | 0.188 | 0.035 |
| WDC | 0.181 | 0.033 |
| STX | 0.177 | 0.031 |
| VZ | 0.165 | 0.027 |
| UNH | 0.156 | 0.024 |
| PG | 0.149 | 0.022 |
| ADI | 0.146 | 0.021 |
| CERN | 0.145 | 0.021 |
| EBAY | -0.095 | 0.009 |
| KO | -0.095 | 0.009 |
| MAT | 0.091 | 0.008 |
| BA | 0.088 | 0.008 |
| VOD | 0.083 | 0.007 |
| DTV | -0.074 | 0.006 |
| MU | -0.072 | 0.005 |
| ORLY | 0.067 | 0.005 |
| CA | 0.042 | 0.002 |
| VIP | 0.029 | 0.001 |
| CAT | -0.023 | 0.001 |
| FAST | 0.022 | 0.000 |
| MMM | 0.016 | 0.000 |
| V | 0.015 | 0.000 |
| DISH | -0.014 | 0.000 |
| T | 0.001 | 0.000 |

Table B. Number of Intensive Search Periods (ISPs) and percent of negative ISPs in each stock ticker.

| *Ticker* | *No. ISPs* | *% Neg* |
| --- | --- | --- |
| CSCO | 47 | 40.4% |
| AAPL | 47 | 34.0% |
| MSFT | 65 | 43.1% |
| INTC | 70 | 42.9% |
| BIDU | 63 | 34.9% |
| JPM | 54 | 48.1% |
| GOOG | 48 | 47.9% |
| WMT | 82 | 47.6% |
| PFE | 111 | 44.1% |
| YHOO | 82 | 39.1% |
| JNJ | 116 | 48.3% |
| XOM | 86 | 45.3% |
| ADP | 69 | 43.8% |

Table C. Correlations between *Absolute Stock Return* and the three search indices in each stock, separately for positive and negative Intensive Search Periods (ISPs).

|  | *ISP Peak* | | | | *ISP Sum* | | | *ISP Duration* | | |  |
| --- | --- | --- | --- | --- | --- | --- | --- | --- | --- | --- | --- |
| *Ticker* | **Positive ISP** | | **Negative ISP** | | **Positive ISP** | **Negative ISP** | | **Positive ISP** | **Negative ISP** | |  |
| CSCO | 0.54 | | 0.55 | | 0.15 | 0.69 | | 0.18 | 0.70 | |  |
| AAPL | 0.01 | | 0.70 | | 0.04 | 0.80 | | 0.04 | 0.52 | |  |
| MSFT | 0.13 | | 0.39 | | 0.006 | 0.40 | | <0.001 | 0.18 | |  |
| INTC | 0.12 | | 0.07 | | 0.08 | 0.03 | | 0.06 | 0.05 | |  |
| BIDU | 0.19 | | 0.55 | | 0.13 | 0.01 | | 0.05 | <0.001 | |  |
| JPM | 0.03 | | 0.18 | | 0.02 | 0.27 | | 0.01 | 0.20 | |  |
| GOOG | 0.57 | | 0.38 | | 0.02 | 0.003 | | 0.005 | 0.009 | |  |
| WMT | 0.005 | | 0.44 | | 0.11 | 0.06 | | 0.11 | 0.04 | |  |
| PFE | 0.21 | | 0.09 | | 0.02 | 0.19 | | 0.002 | 0.15 | |  |
| YHOO | 0.17 | | 0.21 | | 0.009 | <0.001 | | <0.001 | 0.007 | |  |
| JNJ | 0.15 | | 0.31 | | 0.25 | 0.34 | | 0.18 | 0.25 | |  |
| XOM | 0.06 | | 0.09 | | 0.01 | 0.31 | | 0.001 | 0.18 | |  |
| ADP | 0.02 | | 0.03 | | 0.004 | 0.08 | | 0.003 | 0.07 | |  |
| % r^2^(-)>r^2^(+) | | 77% | | 62% | | | 77% | | |  | |

Note: The bottom raw denotes the percentage of tickers where r^2^ was higher for negative ISPs than for positive ISPs. The pattern of higher correlations for negative ISPs was found in the majority of studied stocks.
