# Supplementary material for: Association between Stock Market Gains and Losses and Google Searches
Source: PLoS One. 2015 Oct 29;10(10):e0141354. doi: 10.1371/journal.pone.0141354 (PMC4626086; doi:10.1371/journal.pone.0141354)
Supplement: S2 Text — (DOC) [file pone.0141354.s002.doc]

Association between Stock Market Gains and Losses and Google Searches

S2. Examination of different criteria for Intensive Search Periods

Table A. Regressions of *ISP* *Peak*, *ISP* *Sum*, and *ISP* *Duration* with *Absolute Stock Return* as predictor. The upper threshold, which determines the beginning of the ISP for each stock *i*, is set here to μ*_i_*+0.5*σ*_i_* .

|  | *ISP Peak* | | | *ISP Sum* | *ISP Duration* |
| --- | --- | --- | --- | --- | --- |
| Intercept | | 0.29 | | 0.63 | 1.91 |
| IsNeg | | -0.03 (0.313) | | -0.34  (0.027) | -0.54 (0.004) |
| Absolute Stock Return | | 4.72  (0.001) | | 13.71  (0.002) | 14.68 (0.005) |
| Absolute Stock Return × IsNeg | | 1.42 (0.308) | | 12.99 (0.087) | 16.17  (0.025) |
| F (Model) | | 8.32 (0.003) | | 7.05 (0.006) | 8.80 (0.002) |
| r^2^ | | | 0.15 | 0.07 | 0.04 |

Note: Estimated regression coefficients and p-values in parentheses, followed by the results of the model’s F test and explained variance (r^2^). The sample size was 1870 for all three variables. The dummy variable IsNeg equaled 1 if the daily stock return at the beginning of the period was negative and 0 if not.

Table B. Regressions of *ISP* *Peak*, *ISP* *Sum*, and *ISP* *Duration*, separately for positive and negative Intensive Search Periods. The upper threshold is set here to μ*_i_*+0.5*σ*_i_*.

|  | *ISP Peak* | | *ISP Sum* | | *ISP Duration* | |
| --- | --- | --- | --- | --- | --- | --- |
|  | **Positive**  **ISP** | **Negative**  **ISP** | **Positive**  **ISP** | **Negative**  **ISP** | **Positive**  **ISP** | **Negative**  **ISP** |
| Intercept | 0.29 | 0.26 | 0.62 | 0.28 | 1.91 | 1.37 |
| Absolute Stock Return | 4.72 (0.001) | 6.15 (0.003) | 13.14 (0.002) | 26.13 (0.004) | 14.68 (0.005) | 30.86 (0.001) |
| r^2^ | 0.10 | 0.20 | 0.03 | 0.14 | 0.01 | 0.09 |

Note: Estimated regression coefficients and p-values in parentheses, followed by the explained variance (r^2^). The sample size was 1082 for the positive ISP regressions and 788 for the negative ISP regressions.

Table C. Regressions of *ISP* *Peak*, *ISP* *Sum*, and *ISP* *Duration* with *Absolute Stock Return* as predictor. The upper threshold is set here to μ*_i_*+2*σ*_i_*.

|  | *ISP Peak* | | *ISP Sum* | *ISP Duration* | |
| --- | --- | --- | --- | --- | --- |
| Intercept | | 0.79 | 2.25 | | 4.01 |
| IsNeg | | -0.07 (0.180) | -0.77 (0.097) | | -0.82 (0.220) |
| Absolute Stock Return | | 4.40 (0.007) | 11.08  (0.221) | | 3.84 (0.752) |
| Absolute Stock Return × IsNeg | | 0.99 (0.306) | 15.84 (0.079) | | 16.00 (0.197) |
| F (Model) | | 4.52 (0.024) | 2.06 (0.159) | | 0.94 (0.453) |
| r^2^ | | 0.17 | 0.08 | | 0.02 |

Note: Estimated regression coefficients and p-values in parentheses, followed by the results of the model’s F test and explained variance (r^2^). The sample size was 276 for all three variables. The dummy variable IsNeg equaled 1 if the daily stock return at the beginning of the period was negative and 0 if not.

Table D. Regressions of *ISP* *Peak*, *ISP* *Sum*, and *ISP* *Duration*, separately for positive and negative Intensive Search Periods. The upper threshold is set here to μ*_i_*+2*σ*_i_*.

|  | *ISP Peak* | | *ISP Sum* | | *ISP Duration* | |
| --- | --- | --- | --- | --- | --- | --- |
|  | **Positive**  **ISP** | **Negative**  **ISP** | **Positive**  **ISP** | **Negative**  **ISP** | **Positive**  **ISP** | **Negative**  **ISP** |
| Intercept | 0.79 | 0.72 | 2.25 | 1.48 | 4.01 | 3.19 |
| Absolute Stock Return | 4.40 (0.007) | 5.40 (0.009) | 11.08 (0.220) | 26.92 (0.042) | 3.84  (0.751) | 19.84 (0.159) |
| r^2^ | 0.13 | 0.21 | 0.02 | 0.13 | 0.00 | 0.03 |

Note: Estimated regression coefficients and p-values in parentheses, followed by the explained variance (r^2^). The sample size was 147 for the positive ISP regressions and 129 for the negative ISP regressions.

Table E. Regressions of *ISP* *Peak*, *ISP* *Sum*, and *ISP* *Duration* with *Absolute Stock Return* as predictor. The upper threshold, which determines the beginning of the ISP, is set here to μ*_i_*+3*σ*_i_*.

|  | *ISP Peak* | | *ISP Sum* | *ISP Duration* | |
| --- | --- | --- | --- | --- | --- |
| Intercept | | 1.09 | 2.95 | | 4.16 |
| IsNeg | | 0.00 (0.965) | -0.85 (0.113) | | -1.24 (0.261) |
| Absolute Stock Return | | 1.88 (0. 321) | 6.73 (0.503) | | 14.68 (0.461) |
| Absolute Stock Return × IsNeg | | 1.51 (0.267) | 19.79 (0.087) | | 12.62 (0.555) |
| F (Model) | | 1.31 (0.317) | 2.08 (0.157) | | 2.66 (0.096) |
| r^2^ | | 0.08 | 0.08 | | 0.06 |

Note: Estimated regression coefficients and p-values in parentheses, followed by the results of the model’s F test and explained variance (r^2^). The sample size was 118 for all three variables. The dummy variable IsNeg equaled 1 if the daily stock return at the beginning of the period was negative and 0 if not.

Table F. Regressions of *ISP* *Peak*, *ISP* *Sum*, and *ISP* *Duration*, separately for positive and negative Intensive Search Periods. The upper threshold is set here to μ*_i_*+3*σ*_i_*.

|  | *ISP Peak* | | *ISP Sum* | | *ISP Duration* | |
| --- | --- | --- | --- | --- | --- | --- |
|  | **Positive**  **ISP** | **Negative**  **ISP** | **Positive**  **ISP** | **Negative**  **ISP** | **Positive**  **ISP** | **Negative**  **ISP** |
| Intercept | 1.09 | 1.10 | 2.95 | 2.10 | 4.16 | 2.92 |
| Absolute Stock Return | 1.88 (0.319) | 3.39 (0.093) | 6.73 (0.501) | 26.51 (0.068) | 14.68 (0.459) | 27.30 (0.057) |
| r^2^ | 0.04 | 0.10 | 0.01 | 0.12 | 0.02 | 0.12 |

Note: Estimated regression coefficients and p-values in parentheses, followed by the explained variance (r^2^). The sample size was 62 for the positive ISP regressions and 56 for the negative ISP regressions.
