# Supplementary material for: Association between Stock Market Gains and Losses and Google Searches
Source: PLoS One. 2015 Oct 29;10(10):e0141354. doi: 10.1371/journal.pone.0141354 (PMC4626086; doi:10.1371/journal.pone.0141354)
Supplement: S3 Text — (DOC) [file pone.0141354.s003.doc]

Association between Stock Market Gains and Losses and Google Searches

S3. Examination of differences between correlations in randomly permuted conditions

To examine the robustness of the differences between correlations for positive and negative ISPS, observed in Tables 2 and 4, we conducted a permutations test wherein we examine correlations for randomly set conditions within the current data. First, we created 5,000 exact copies of the original data for each of the dependent variables (total of 25K series). Next, the condition label (positive vs. negative ISP) was randomly shuffled in each individual series. Then we calculated the distribution of the difference between r^2^(-) - r^2^(+) as well as the absolute difference |r^2^(-) - r^2^(+)| in each series, where r^2^(-) and r^2^(+) are the r^2^ values for negative and positive periods, respectively. This was carried out separately for each of the five dependent measures (i.e., in each of 5,000 series). These differences and absolute differences are shown in Table A below. As can be seen they were far smaller than the actual difference in r^2^ between conditions (appearing on the right hand column of the table). This indicates that the effect of valence (calculated through the ANOVAs) was not a by-product of the particular distribution of the current data.

Table A. Mean difference and absolute difference in r^2^ between randomly permuted conditions.

|  | | Randomly permuted data | | Actual data |
| --- | --- | --- | --- | --- |
|  | | r^2^(-) - r^2^(+) | |r^2^(-) - r^2^(+)| | r^2^(-) - r^2^(+) |
| ISP Peak | 0.0008 (0.0011) | | 0.06 (0.0013) | 0.19 |
| ISP Sum | 0.0003 (0.0006) | | 0.04  (0.0009) | 0.15 |
| ISP Duration | -0.0008 (0.0008) | | 0.03 (0.0005) | 0.08 |
| ISP Post-Peak Sum | -0.0003  (0.0004) | | 0.03  (0.0005) | 0.08 |
| ISP Post-Peak Duration | -0.0008  (0.0004) | | 0.02  (0.0004) | 0.05 |

Note: The numbers in parentheses denote the confidence interval in the simulation. The right column presents the actual difference between r^2^s (extracted from Tables 2 and 4 in the main text).
